# Smart management of single surgical instruments through unique device identification (UDI) barcode tracking system for enhancing sterilization quality and patient safety

**DOI:** 10.1017/ash.2025.101

**Published:** 2025-09-03

**Authors:** Jr-Huei Liu, Chu-Ying Wu, Wei-Ting Liang, Min-Fen Hsu, Chiu-Yu Liu

**Affiliations:** 1 Pingtung Veterans General Hospital Nursing Department; 2 Meiho University; 3 Central Sterile Supply Department; 4 Operating Room

## Abstract

**Introduction:** We propose to develop a Unique Device Identification (UDI) barcode tracking system for surgical instruments. This system aims to enhance hospital processes, thereby benefiting both patients and staff members. **Methods:** The UDI barcode tracking system for surgical instruments was implemented in March 2023: 1. Each surgical instrument underwent laser engraving with a UDI barcode, encompassing relevant data such as instrument name, image, model, specifications, origin, license, Instructions for Use (IFU), and total distribution quantity. 2. Upon scanning the engraved serial number, the system automatically discerns whether the instrument belongs to the designated set. 3. Mechanical, chemical, and biological monitoring indicators are integrated into the tracking system, with automatic adjudication for release into storage if criteria are met; otherwise, notifications are issued for review and retrieval by personnel. **Results:** 1. Between March 2023 and February 2024, a total of 157,614 instrument sets were equipped with this system, enabling staff to achieve a zero-error rate in rapid and precise instrument identification. 2.During this period, 4,026 cycles of high-temperature sterilization monitoring and 380 cycles of low- temperature H2O2 plasma sterilization monitoring were recorded. 3.Each monitoring cycle was digitally recorded, obviating the necessity for paper-based documentation and saving a total of 4,406 A4 paper sheets. 4. In the same timeframe, a total of 85,899 packages were dispensed, each linked to patient medical record numbers. **Conclusions:** The adoption of the surgical instrument UDI barcode tracking system by our institution’s central sterilization supply department has garnered participation from 622 individuals. It not only reduces the time spent by staff searching for items and conducting educational training but also automatically identifies whether the instrument belongs to the package, thereby enhancing inventory efficiency and reducing the incidence of errors. Sterilization monitoring indicators are automatically uploaded and intercepted to uphold patient safety.

